# Mammographic densities as a criterion for entry to a clinical trial of breast cancer prevention.

**DOI:** 10.1038/bjc.1995.358

**Published:** 1995-08

**Authors:** N. F. Boyd, E. Fishell, R. Jong, J. C. MacDonald, R. K. Sparrow, I. S. Simor, V. Kriukov, G. Lockwood, D. Tritchler

**Affiliations:** Division of Epidemiology and Statistics, Ontario Cancer Institute, Toronto, Canada.

## Abstract

The most convincing evidence that a factor such as dietary fat is causally related to breast cancer would be obtained from a randomised controlled trial in which exposure to dietary fat intake was systematically varied. A limitation of randomised controlled trials of breast cancer prevention, however, is the large sample size required to detect plausible reductions in risk resulting from the intervention. We describe here experience over a period of 9 years with the use of one risk factor for breast cancer as a criterion for entry to a clinical trial of breast cancer prevention. The risk factor used was the presence of extensive densities in the breast tissue on mammography, which has been found by several investigators to be strongly associated with risk of breast cancer. Using this criterion for selection, 1800 subjects of mean age 46 years were enrolled between 1982 and 1986, and again between 1988 and the present. Throughout this period, the point estimate of annual invasive cancer incidence was approximately 6 per 1000 per year. The observed cancer incidence has been consistently 4-5 times the incidence expected from age-specific breast cancer incidence data for women living in Ontario. These data show that the selection of subjects for a clinical trial of breast cancer prevention using the criterion of extensive breast parenchymal densities does identify a group at substantially increased risk of breast cancer. Use of this criterion for the selection of subjects can substantially reduce the sample size required for a clinical trial of a preventive strategy.


British Journal of Cancer (1995) 72, 476-479
© 1995 Stockton Press All rights reserved 0007-0920/95 $12.00

### Mammographic densities as a criterion for entry to a clinical trial of breast cancer prevention

NF Boyd[1,2], E Fishell[3], R Jong[4], JC MacDonald[5], RK Sparrow[6], IS Simor[4], V Kriukov[1], G Lockwood[1] and D Tritchler[1]

[1]*Division of Epidemiology and Statistics, Ontario Cancer Institute;* [2]*Division of Preventive Oncology, Ontario Cancer Treatment and Research Foundation;* [3]*Department of Diagnostic Imaging, Women's College Hospital;* [4]*Department of Radiological Sciences, Mount Sinai Hospital;* [5]*Department of Diagnostic Imaging, Windsor Western Hospital;* [6]*Department of Diagnostic Imaging, Victoria Hospital, Canada.*

**Summary** The most convincing evidence that a factor such as dietary fat is causally related to breast cancer would be obtained from a randomised controlled trial in which exposure to dietary fat intake was systematically varied. A limitation of randomised controlled trials of breast cancer prevention, however, is the large sample size required to detect plausible reductions in risk resulting from the intervention. We describe here experience over a period of 9 years with the use of one risk factor for breast cancer as a criterion for entry to a clinical trial of breast cancer prevention. The risk factor used was the presence of extensive densities in the breast tissue on mammography, which has been found by several investigators to be strongly associated with risk of breast cancer. Using this criterion for selection, 1800 subjects of mean age 46 years were enrolled between 1982 and 1986, and again between 1988 and the present. Throughout this period, the point estimate of annual invasive cancer incidence was approximately 6 per 1000 per year. The observed cancer incidence has been consistently 4-5 times the incidence expected from age-specific breast cancer incidence data for women living in Ontario. These data show that the selection of subjects for a clinical trial of breast cancer prevention using the criterion of extensive breast parenchymal densities does identify a group at substantially increased risk of breast cancer. Use of this criterion for the selection of subjects can substantially reduce the sample size required for a clinical trial of a preventive strategy.

**Keywords:** breast cancer; mammographic densities; breast cancer risk

Breast cancer is a major cause of morbidity and mortality throughout most of the Western world, and therapeutic progress against the disease has been slow (Bailar and Smith, 1986). There is, however, epidemiological evidence indicating that the disease can be prevented (Doll and Peto, 1981). Wide variations in breast cancer incidence and mortality between countries, and changes in disease rates in migrants, clearly indicate that environmental factors play a role in causing breast cancer, and suggest that reducing exposure to these factors might lead to a reduction in risk of the disease. Several environmental factors which might vary between individuals, such as body weight (London *et al*., 1989; Baanders and de Waard, 1992); and consumption of alcohol (Longnecker *et al*., 1988), dietary fat (Prentice and Sheppard, 1990; Willett and Stampfer, 1990; Hiller and McMichael 1990; Howe, 1990), fibre (Rose, 1990) and antioxidant vitamins (Block 1991), have been described as being associated with breast cancer risk. However, it is not yet clear that any of these factors is causally related to breast cancer and there is controversy about the role of several of them.

The most convincing evidence that an environmental factor is causally related to breast cancer, and that changing exposure to it would reduce the risk of breast cancer, would be obtained from a randomised controlled trial (Prentice *et al*., 1988; Boyd *et al*., 1990*a*). In such a trial, exposure to the putative causative factor would be reduced or eliminated (or increased in the case of fibre and antioxidant vitamins) in a randomly selected group of women, and these subjects, together with a control group, observed for the development of tumours. Trials involving dietary and pharmacological interventions in the prevention of breast cancer are now in progress.

Correspondence: NF Boyd, Division of Epidemiology and Statistics, Ontario Cancer Institute, 500 Sherbourne Street, Toronto, Ontario M4X 1K9, Canada
Received 22 July 1994; revised 20 February 1995; accepted 30 March 1995

A limitation of randomised controlled trials of breast cancer prevention, however, is the extremely large sample size required to detect plausible reductions in risk resulting from the intervention. The sample size of such trials is influenced by a number of factors, including the expected cancer incidence in the absence of the intervention, the duration of the trial and the extent to which the intervention is expected to reduce risk (Self *et al*., 1988). For any given postulated risk reduction and trial duration, the higher the risk of disease in the control group, the smaller, and less expensive, will be the trial.

We describe here experience over a period of 9 years with the use of one risk factor for breast cancer as a criterion for entry to a clinical trial of breast cancer prevention. The risk factor used was the presence of extensive densities in the breast parenchyma on mammography, which has been found by several investigators to be strongly associated with risk of breast cancer (Wolfe, 1976*a*,*b*; Saftlas and Szklo, 1987; Oza and Boyd, 1993).

### Materials and methods

### *Selection of subjects*

After completing work on the relationship of dense breast parenchyma to breast cancer risk (Boyd *et al*., 1982*a*, 1982*b*), we began in 1982 to recruit women with mammographic parenchymal densities in at least 50% of the breast into a series of trials testing a dietary intervention. Between 1982 and 1986, 295 women with these radiological characteristics were recruited into pilot studies (Boyd *et al*., 1988), and in 1988 a randomised clinical trial was started to determine if breast cancer incidence could be reduced by the dietary intervention developed in these pilot studies (Boyd *et al*., 1990). Recruitment for this trial was expanded, and a total of 2040 subjects have been enrolled to date. The total number of subjects recruited throughout this period is therefore approximately 2335.

*Brief description of the intervention*

All subjects recruited were, after initial assessment, randomly allocated to receive one of two types of dietary advice. A group of controls received teaching according to current government guidelines (Health and Welfare, Canada, 1992). These provide general advice about healthy eating, but did not, until 1993, specify a desirable level of dietary fat intake. The members of this group were not counselled to change their intake of dietary fat. An intervention group received teaching and dietary counselling designed to reduce dietary fat intake to a target level of 15% of total calories. Further details concerning the intervention and the dietary and other changes that result from it have been given elsewhere (Boyd *et al.*, 1988*a*,*b*, 1990*b*, 1992).

*Follow-up*

Subjects enrolled between 1982 and 1986 took part in pilot studies of 12–24 months' duration. Since 1986 all subjects who participated in these pilot studies have been contacted annually and asked to respond to a short questionnaire about breast biopsies during the previous 12 months. Subjects entered into the cancer prevention trial since 1988 have been asked to respond to the same questionnaire administered at the clinic visit closest to the anniversary of the date of randomisation. Subjects who drop out of the trial by no longer attending clinic visits are contacted annually by mail and/or telephone. For those who report having had breast biopsies, permission is sought to obtain pathology reports and histological slides from the hospitals concerned. In addition to contacting all of those in the cancer prevention trial, we have been successful in contacting annually 95% of the 295 subjects from pilot studies and 90% of the subjects who have dropped out of the trial.

### Results

*Characteristics of subjects*

Table I shows selected characteristics separately for subjects enrolled between 1982 and 1986, and since 1988. Subjects in these two time periods are similar with respect to height, mean age at first pregnancy and marital status. A family history of breast cancer in at least one first-degree relative was found in just under 20% of the subjects in both groups. The subjects enrolled since 1988 were more often parous and had slightly more children. They also were somewhat heavier and approximately 2 years older. These differences reflect the expansion of the sample population from Toronto to smaller cities, London, Windsor and Hamilton, and from diagnostic clinics to screening centres.

*Cancer risk*

Figure 1 shows the cumulative incidence of invasive cancer (i.e. cancers developing after randomisation) in the total population of subjects described in Table I and includes all members of both intervention and control groups combined as it is too early in the trial to consider an analysis according to randomisation. The cumulative invasive cancer incidence has been calculated on 1 January for each of the years shown, and the point estimates of risk, calculated as an annual rate per 1000 person–years of observation, and the associated 95% confidence intervals calculated. Also, at each time period, we have calculated the annual incidence of invasive breast cancer expected based upon the age distribution of the trial population, the length of the period of observation, and age-specific rates for the Ontario population. The expected rates for each time interval are shown in the figures as asterisks.

From 1986, when cancer risk was first calculated for subjects enrolled in pilot studies, to 1995, the point estimate of cancer risk is approximately 6 per 1000 per year. At each of these intervals the observed cancer incidence exceeds the incidence expected in the population of Ontario. The incidence observed has been consistently 4–5 times the incidence expected from age-specific breast cancer incidence data for women living in Ontario. Estimates of risk from subjects in both the pilot studies and the main cancer prevention trial, over the entire period of observation, are identical and are both 4.5 times the age-specific risk for the population (data not shown).

**Table I** Selected characteristics of study participants

| *Characteristics* | *1982–86* | *1988 to present* | P-*value*[a] |
|---|---|---|---|
| Number | 295 | 2,003 | |
| Mean age (years) | 43.8 | 46.0 | <0.01 |
| Mean height (cm) | 163.2 | 163.2 | 0.99 |
| Mean weight (kg) | 59.6 | 62.8 | <0.01 |
| Premenopausal (%) | 76.9 | 74.1 | 0.30 |
| Post-menopausal (%) | 23.1 | 25.9 | |
| Mean age at first pregnancy | 25.1 | 25.6 | 0.18 |
| Parous (%) | 63.9 | 71.8 | 0.01 |
| Mean number of children | 1.4 | 1.6 | 0.01 |
| First-degree relatives with breast cancer (%) | 18.4 | 18.9 | 0.82 |

[a]Continuous variables compared using *t*-tests; category variables compared using 2 × 2 chi-square tests.

**Figure 1** (**a**) Cancer risk per 1000 person–years. (**b**) Cancer risk per 1000 person–years excluding cancer in the first 12 months. (**c**) Cancer risk per 1000 person–years excluding cancer in the first 24 months.

Table II Observed and expected risk of breast cancer according to year

| Year | PY | Cancers | Obs | 95% CI | Exp | Obs/Exp |
|---|---|---|---|---|---|---|
| 1986 | 688 | 4 | 5.81 | 2.18–15.48 | 1.29 | 4.5 |
| 1987 | 977 | 6 | 6.14 | 2.76–13.67 | 1.31 | 4.69 |
| 1988 | 1272 | 7 | 5.5 | 2.62–11.54 | 1.34 | 4.1 |
| 1989 | 1617 | 9 | 5.56 | 2.82–10.69 | 1.36 | 4.09 |
| 1990 | 2139 | 14 | 6.55 | 3.9–11.06 | 1.39 | 4.71 |
| 1991 | 2845 | 17 | 5.98 | 3.72–9.62 | 1.41 | 4.24 |
| 1992 | 3771 | 23 | 6.1 | 4.05–9.18 | 1.43 | 4.27 |
| 1993 | 5143 | 31 | 6.02 | 4.23–8.54 | 1.45 | 4.15 |
| 1994 | 7004 | 36 | 5.14 | 3.71–7.14 | 1.47 | 3.5 |
| 1995 | 9174 | 45 | 4.9 | 3.66–6.56 | 1.5 | 3.27 |

PY, person–years; Obs, observed cancer incidence per 1000 PY; Exp, expected cancer incidence per 1000 PY.

To examine the possibility that the 'masking' of breast cancer by dense breast parenchyma at the time of entry contributed to the increased cancer incidence observed (Egan and Mosteller, 1977; Whitehead *et al.*, 1985; Ma *et al.*, 1992), we recalculated cancer risk for each interval after first excluding all cancers occurring within 12 months, and then within 24 months, of randomisation. The results shown in Figure 1 show an annual risk that is essentially the same as those shown before exclusions in Figure 1, although the confidence intervals are of course wider.

### Discussion

These data show that the selection of subjects for a clinical trial of breast cancer prevention using the criterion of extensive breast parenchymal densities does identify a group at substantially increased risk of breast cancer. The risk experienced by this group is approximately 4–5 times that of the general population of the same age and has now been observed consistently over a period of 9 years. The 95% confidence interval associated with the most recent estimate of risk shows that we can now exclude a risk lower than 2.9 times that of the age-specific rate for the population. The results do not appear to be explained, or even influenced, by 'masking' (Egan and Mosteller, 1977; Whitehead *et al.*, 1985; Ma *et al.*, 1992), that is the presence of undetected cancer at the time of recruitment.

These findings are consistent with other epidemiological data showing that extensive mammographic densities are a risk factor for the development of breast cancer (Saftlas and Szklo, 1987; Oza and Boyd, 1993). This evidence has been comprehensively reviewed and shows that most well-conducted epidemiological studies, both cohort and case–control in design, have found an association between mammographically dense breast tissue and increased risk of breast cancer. Various methods have been used in the literature for classifying these appearances of breast densities. In examining the effect that the method of classification has on the estimation of cancer risk, we have found quantitative methods that attempt to determine the proportion of the breast occupied by densities generate the highest estimates of risk (Warner *et al.*, 1992). Similar quantitative methods were used to select the subjects described here, and the risk observed is consistent with the high risks predicted from the other papers describing the use of quantitative methods to predict risk (Boyd *et al.*, 1982*b*; Brisson *et al.*, 1982, 1984; Wolfe *et al.*, 1987; Saftlas *et al.*, 1989, 1991).

It is, however, possible that other factors associated with the willingness of subjects to enter a trial of cancer prevention may also have influenced the observed breast cancer risk. The high prevalence of subjects with at least one first-degree relative with breast cancer is one identifiable feature in subjects who entered the trial that may have contributed to the increased risk observed. However, any contribution of this feature to the cancer risk in the present population is likely to be small (Kelsey, 1993).

These findings indicate that the strategy used here to select subjects for a trial of breast cancer prevention can substantially increase the number of cancers expected, and reduce the number of subjects required, compared with a trial that enrols members of the general population of the same age, thus making cancer prevention studies feasible. The frequency of extensive mammographic densities varies with the age of the population examined, but approximately 20% of the participants in the Canadian National Breast Screening Trial, a randomised trial of mammographic screening in women aged 40–59, had more than 50% of the breast occupied by mammographic densities and would have been eligible for this cancer prevention trial.

We have used the statistical procedure described by Self *et al.* (1988) to determine the number of subjects required for a clinical trial of breast cancer prevention, making the same assumptions about the difference in dietary fat intake between control and intervention subjects and the extent to which cancer rates might be influenced by such a fat differential, but different assumptions about rates of breast cancer in the control group. We find that a total of 6000 subjects is required using the estimates of cancer risk described in the present paper, compared with a total sample size of 26 000 if age-specific cancer rates for the Ontario population are used.

The use of a criterion such as mammographic densities for selecting subjects for breast cancer prevention studies raises questions about the generalisability of the results to individuals with other mammographic characteristics. We have found in other studies that mammographic densities in more than 50% of the breast are present in a substantial proportion of women who develop breast cancer. For example, in the Canadian National Breast Screening Study, 44% of those who developed breast cancer had at entry to the trial mammographic densities that would have made them eligible for our present trial (Boyd *et al.*, 1994). Further, mammographic densities are a continuous variable that influence risk of breast cancer throughout the range of measurement, with a 2% increase in the relative risk of breast cancer for each 1% increase in density (Boyd *et al.*, 1994). Use of the criterion of 50% density in breast tissue as a criterion for trial eligibility therefore selects subjects in the upper part of a continuum of risk. There is currently no evidence to indicate that mammographic densities are directly related to diet, or that the results of dietary change in the group of individuals selected for the trial described here will differ qualitatively from those seen in women at average risk of breast cancer. Any result found in high-risk subjects is likely to apply also to those of average risk. The extent to which women at average risk of breast cancer will be prepared to alter their diets is uncertain, but presumably their willingness to change will be influenced by the results of trials such as the one described here.

**Acknowledgements**
This study was supported by grants from the Ontario Ministry of Health, the Medical Research Council of Canada and the Canadian Breast Cancer Research Initiative.

### References

BAANDERS AN AND DE WAARD F. (1992). Breast cancer in Europe: the importance of factors operating at an early age. *Eur. J. Cancer Prev.*, **1**, 285–291.

BAILAR JC AND SMITH EM. (1986). Progress against cancer? *N. Engl. J. Med.*, **314**, 1226–1232.

BLOCK G. (1991). Vitamin C and cancer prevention: the epidemiologic evidence. *Am. J. Clin. Nutr.*, **53**, 270s–282s.

BOYD NF, O'SULLIVAN B, CAMPBELL JE, FISHELL E, SIMOR I, COOKE G AND GERMANSON T. (1982*a*). Bias and the association of mammographic parenchymal patterns with breast cancer. *Br. J. Cancer*, **45**, 179–184.

BOYD NF, O'SULLIVAN B, CAMPBELL JE, FISHELL E, SIMOR I, COOKE G AND GERMANSON T. (1982*b*). Mammographic signs as risk factors for breast cancer. *Br. J. Cancer*, **45**, 185–193.

BOYD NF, COUSINS M, BEATON M, FISHELL E, WRIGHT B, FISH E, KRIUKOV V, LOCKWOOD G, TRITCHLER D, HANNA W AND PAGE DL. (1988*a*). A clinical trial of a low fat, high carbohydrate diet in patients with mammographic dysplasia: report of early outcomes. *J. Natl Cancer Inst.*, **80**, 1244–1248.

BOYD NF, MCGUIRE V, SHANNON P, COUSINS M, KRIUKOV V, MAHONEY L, FISH E, LICKLEY L, LOCKWOOD G AND TRITCHLER D. (1988*b*). The effect of a low fat, high carbohydrate diet on symptoms of cyclical mastopathy. *Lancet*, **2**, 128–132.

BOYD NF, COUSINS M, LOCKWOOD, G. & TRITCHLER, D. (1990*a*). The feasibility of testing experimentally the dietary fat–breast cancer hypothesis. *Br. J. Cancer*, **62**, 878–881.

BOYD NF, COUSINS M, BEATON M, KRIUKOV V, LOCKWOOD G AND TRITCHLER D. (1990*b*). Relationship between dietary change and serum cholesterol in women: results from a randomized controlled trial. *Am. J. Clin. Nutr.*, **52**, 470–476.

BOYD NF, COUSINS M AND KRIUKOV V. (1992). A randomized controlled trial of a low fat diet: Retention of subjects and characteristics of drop outs. *J. Clin. Epidemiol.*, **45**, 31–38.

BOYD NF, BYNG J, YAFFE M, JONG R, FISHELL E, LITTLE L, MILLER AB, LOCKWOOD G, TRITCHLER D. (1994). Quantitative classification of mammographic densities and breast cancer risk: results from the Canadian National Breast Screening study. *J. Natl Cancer Inst.*, **87**, 670–675.

BRISSON J, SADOWSKY NL, TWADDLE JA, MORRISON AS, COLE P, MERLETTI F. (1982). The relation of mammographic features of the breast to breast cancer risk factors. *Am. J. Epidemiol.*, **115**, 438–443.

BRISSON J, MORRISON AS, KOPANS DB, SADAWSKY NL, KALISHER T, TWADDLE JA, MEYER JE, HENSCHKE CI, COLE P. (1984). Height and weight, mammographic features of breast tissue, and breast cancer risk. *Am. J. Epidemiol.*, **119**, 371–381.

DOLL R AND PETO R. (1981). *The Causes of Cancer: Quantitative Estimates of Avoidable Risks of Cancer in the United States.* Oxford University Press: Oxford.

EGAN RL AND MOSTELLER RC. (1977). Breast cancer mammography patterns. *Cancer*, **45**, 2550–2556.

HEALTH AND WELFARE, CANADA. (1992). In *Canada's Food Guide to Healthy Eating*. Minister of Supplies and Services: Canada.

HILLER JE AND MCMICHAEL AJ. (1990). Dietary fat and cancer: a comeback for ecological studies? *Cancer Causes Control*, **1**, 101–102.

HOWE GR. (1990). Dietary fat and cancer. *Cancer Causes Control*, **1**, 99–100.

KELSEY JL. (1993). Breast cancer epidemiology: summary and future directions. *Epidemiol. Rev.*, **15**, 256–263.

LONDON SJ, COLDITX GA, STAMPFER MJ, WILLETT WC, ROSNER B AND SPEIZER FE. (1989). Prospective study of relative weight, height and risk of breast cancer. *JAMA*, **262**, 2853–2858.

LONGNECKER MP, BERLIN JA, ORZA MJ, CHALMERS TC. (1988). A meta-analysis of alcohol consumption in relation to risk of breast cancer. *JAMA*, **260**, 652–656.

MA L, FISHELL E, WRIGHT B, HANNA W, ALLAN S AND BOYD NF. (1992). A controlled study of factors associated with failure to detect breast cancer by mammography. *J. Natl Cancer Inst.*, **84**, 781–785.

OZA AM AND BOYD NF. (1993). Mammographic parenchymal patterns: A marker of breast cancer risk. *Epidemiol. Rev.*, **15**, 196–208.

PRENTICE RL AND SHEPPARD L. (1990). Dietary fat and cancer: consistency of the epidemiologic data, and disease prevention that may follow from a practical reduction in fat consumption. *Cancer Causes Control*, **1**, 81–97.

PRENTICE RL, KAKAR F, HURSTING S, SHEPPARD L, KLEIN R AND KUSHI LH. (1988). Aspects of the rationale for the women's health trial. *J. Natl Cancer Inst.*, **80**, 802–814.

ROSE DP. (1990). Dietary fat and breast cancer. *Nutr. Cancer*, **13**, 1–8.

SAFTLAS A AND SZKLO M. (1987). Mammographic parenchymal patterns and breast cancer risk. *Epidemiol. Rev.*, **9**, 146–174.

SAFTLAS AF, WOLFE JN, HOOVER RN, BRINTON LA, SCHAIRER C, SALANE M AND SZKLO M. (1989). Mammographic parenchymal patterns as indicators of breast cancer risk. *Am. J. Epidemiol.*, **129**, 518–526.

SAFTLAS AF, HOOVER RN, BRINTON LA, SZKLO M, SALANE M, OLSON DR AND WOLFE JN. (1991). Mammographic densities and risk of breast cancer. *Cancer*, **67**, 2833–2838.

SELF S, PRENTICE R, IVERSON D, HENDERSON M, THOMPSON D, BYAR D, INSULL W, GORBACH SL, CLIFFORD C, GOLDMAN S, URBAN N, SHEPPARD L AND GREENWALD P. (1988*a*). Statistical design of the Women's Health Trial. *Controlled Clin. Trials*, **9**, 119–136.

WARNER E, LOCKWOOD G, TRITCHLER D AND BOYD NF. (1992). The risk of breast cancer associated with mammographic parenchymal patterns: a meta-analysis of the published literature to examine the effect of method of classification. *Cancer Detect. Prev.*, **16**, 67–72.

WHITEHEAD J, CARLILE T, KOPECKY KJ, THOMPSON DJ, GILBERT Jr FI, PRESENT AJ, THREATT BA, KROOK P AND HADAWAY E. (1985). Wolfe mammographic parenchymal patterns. A study of the masking hypothesis of Egan and Mosteller. *Cancer*, **56**, 1280–1286.

WILLETT WC AND STAMPFER MJ. (1990). Dietary fat and cancer: another view. *Cancer Causes Control*, **1**, 103–109.

WOLFE JN. (1976*a*). Risk for breast cancer development determined by parenchymal pattern. *Cancer*, **37**, 2486–2492.

WOLFE JN. (1976*b*). Breast patterns as an index of risk for developing breast cancer. *Am. J. Roentgenol.*, **126**, 1130–1139.

WOLFE JN, SAFTLAS AF AND SALANE M. (1987). Mammographic parenchymal patterns and quantative evaluation of mammographic densities: a case–control study. *Am. J. Roentgenol.*, **148**, 1087–1092.


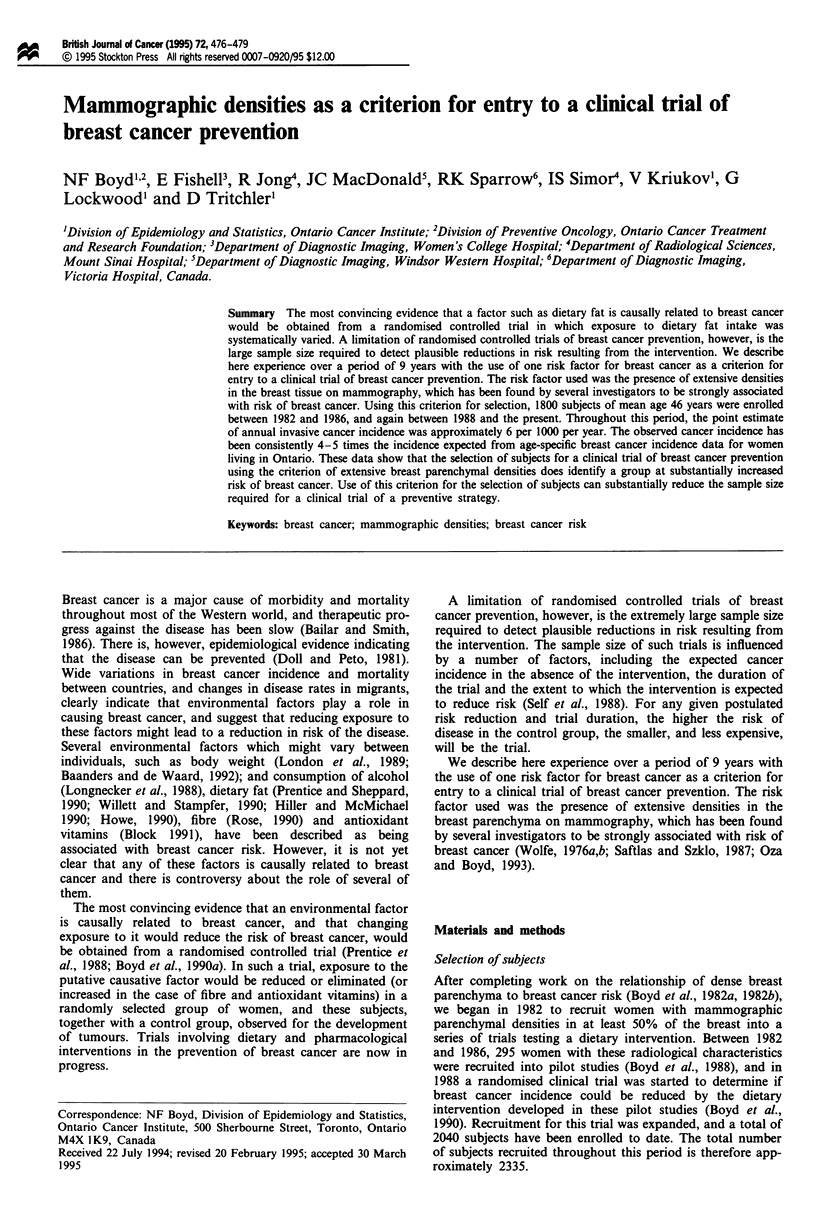

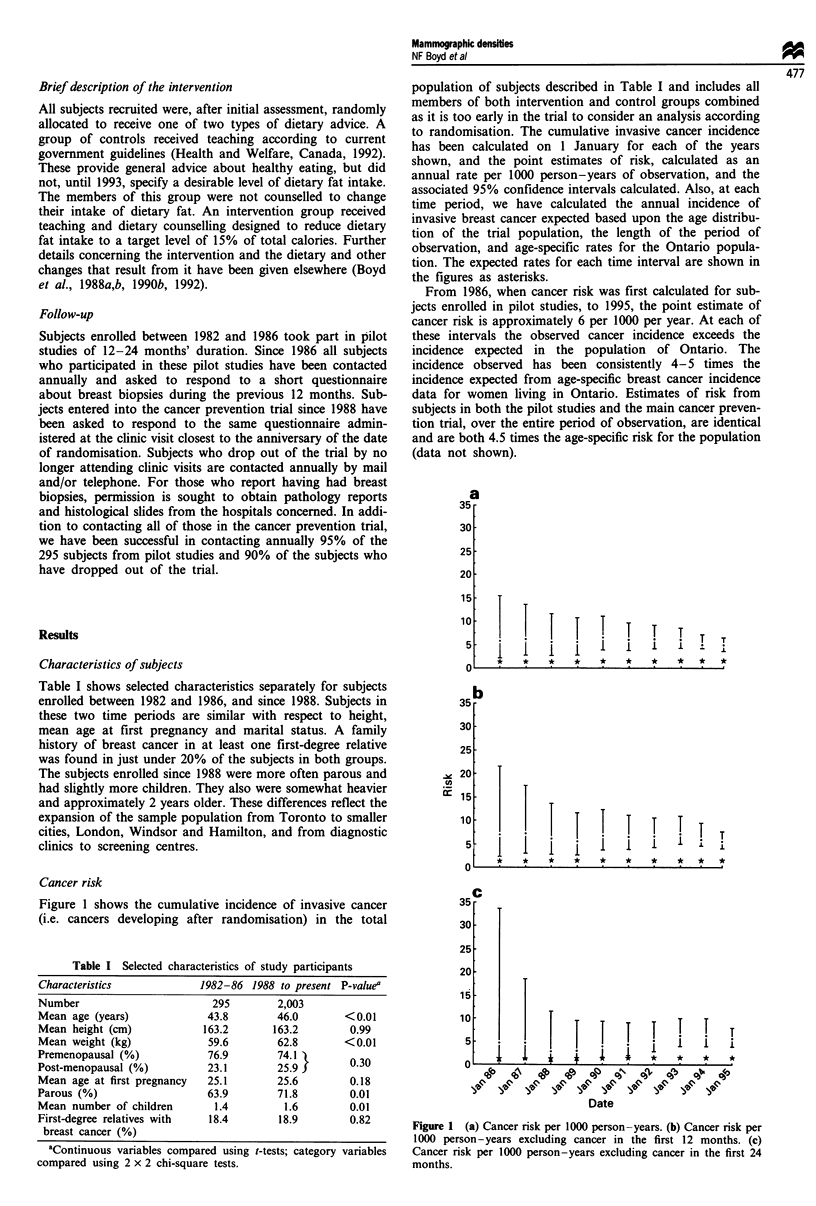

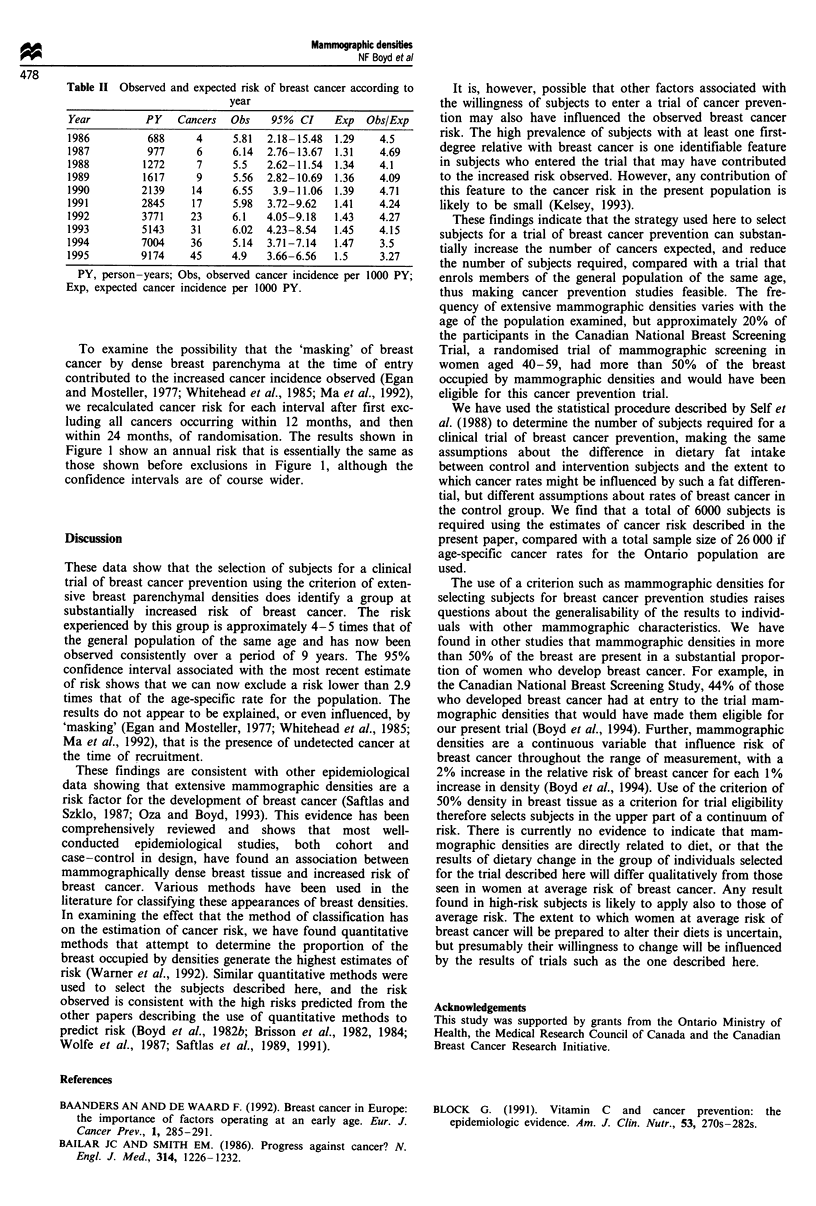

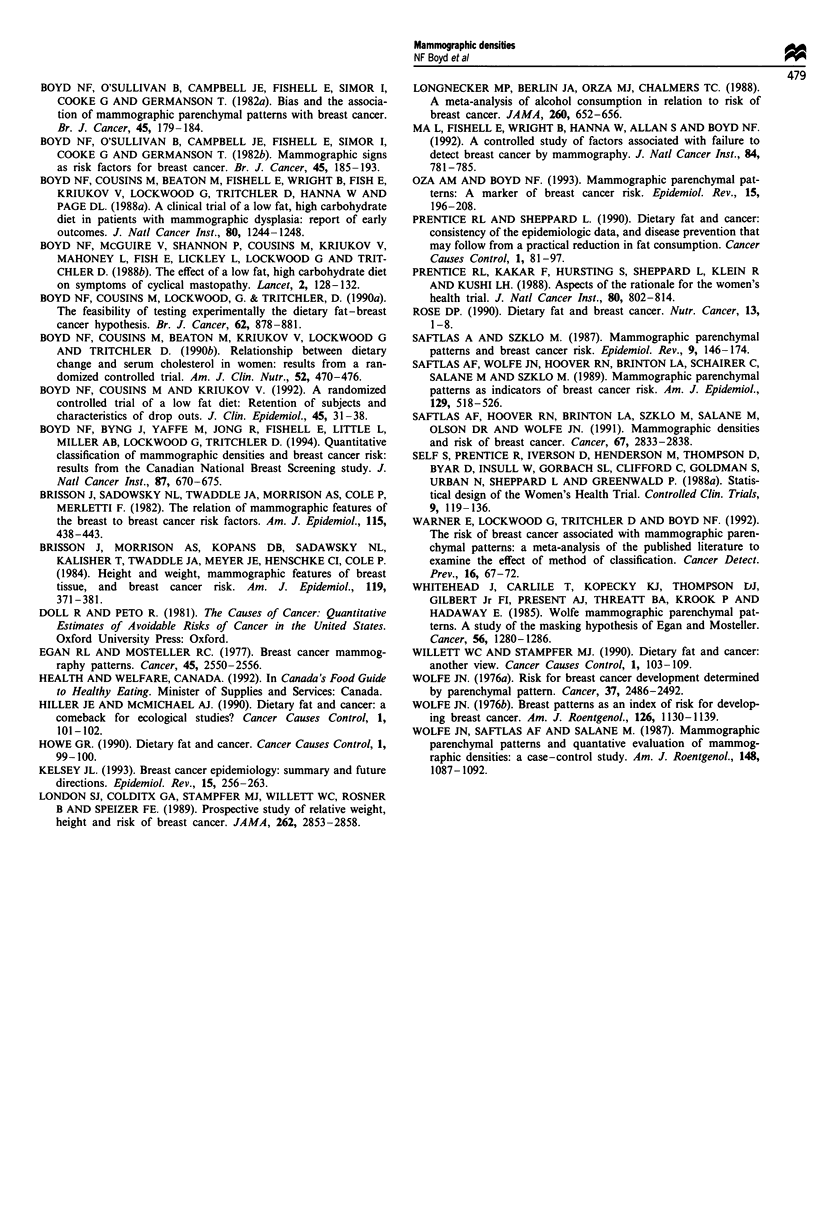


## References

[OCR_00481] Baanders A. N., de Waard F. (1992). Breast cancer in Europe: the importance of factors operating at an early age.. Eur J Cancer Prev.

[OCR_00488] Bailar J. C., Smith E. M. (1986). Progress against cancer?. N Engl J Med.

[OCR_00492] Block G. (1991). Vitamin C and cancer prevention: the epidemiologic evidence.. Am J Clin Nutr.

[OCR_00542] Boyd N. F., Byng J. W., Jong R. A., Fishell E. K., Little L. E., Miller A. B., Lockwood G. A., Tritchler D. L., Yaffe M. J. (1995). Quantitative classification of mammographic densities and breast cancer risk: results from the Canadian National Breast Screening Study.. J Natl Cancer Inst.

[OCR_00512] Boyd N. F., Cousins M., Beaton M., Fishell E., Wright B., Fish E., Kriukov V., Lockwood G., Tritchler D., Hanna W. (1988). Clinical trial of low-fat, high-carbohydrate diet in subjects with mammographic dysplasia: report of early outcomes.. J Natl Cancer Inst.

[OCR_00530] Boyd N. F., Cousins M., Beaton M., Kriukov V., Lockwood G., Tritchler D. (1990). Quantitative changes in dietary fat intake and serum cholesterol in women: results from a randomized, controlled trial.. Am J Clin Nutr.

[OCR_00536] Boyd N. F., Cousins M., Kriukov V. (1992). A randomized controlled trial of dietary fat reduction: the retention of subjects and characteristics of drop outs.. J Clin Epidemiol.

[OCR_00523] Boyd N. F., Cousins M., Lockwood G., Tritchler D. (1990). The feasibility of testing experimentally the dietary fat-breast cancer hypothesis.. Br J Cancer.

[OCR_00520] Boyd N. F., McGuire V., Shannon P., Cousins M., Kriukov V., Mahoney L., Fish E., Lickley L., Lockwood G., Tritchler D. (1988). Effect of a low-fat high-carbohydrate diet on symptoms of cyclical mastopathy.. Lancet.

[OCR_00501] Boyd N. F., O'Sullivan B., Campbell J. E., Fishell E., Simor I., Cooke G., Germanson T. (1982). Bias and the association of mammographic parenchymal patterns with breast cancer.. Br J Cancer.

[OCR_00507] Boyd N. F., O'Sullivan B., Campbell J. E., Fishell E., Simor I., Cooke G., Germanson T. (1982). Mammographic signs as risk factors for breast cancer.. Br J Cancer.

[OCR_00552] Brisson J., Morrison A. S., Kopans D. B., Sadowsky N. L., Kalisher L., Twaddle J. A., Meyer J. E., Henschke C. I., Cole P. (1984). Height and weight, mammographic features of breast tissue, and breast cancer risk.. Am J Epidemiol.

[OCR_00548] Brisson J., Sadowsky N. L., Twaddle J. A., Morrison A. S., Cole P., Merletti F. (1982). The relation of mammographic features of the breast to breast cancer risk factors.. Am J Epidemiol.

[OCR_00581] Kelsey J. L. (1993). Breast cancer epidemiology: summary and future directions.. Epidemiol Rev.

[OCR_00585] London S. J., Colditz G. A., Stampfer M. J., Willett W. C., Rosner B., Speizer F. E. (1989). Prospective study of relative weight, height, and risk of breast cancer.. JAMA.

[OCR_00590] Longnecker M. P., Berlin J. A., Orza M. J., Chalmers T. C. (1988). A meta-analysis of alcohol consumption in relation to risk of breast cancer.. JAMA.

[OCR_00597] Ma L., Fishell E., Wright B., Hanna W., Allan S., Boyd N. F. (1992). Case-control study of factors associated with failure to detect breast cancer by mammography.. J Natl Cancer Inst.

[OCR_00601] Oza A. M., Boyd N. F. (1993). Mammographic parenchymal patterns: a marker of breast cancer risk.. Epidemiol Rev.

[OCR_00615] Prentice R. L., Kakar F., Hursting S., Sheppard L., Klein R., Kushi L. H. (1988). Aspects of the rationale for the Women's Health Trial.. J Natl Cancer Inst.

[OCR_00634] Saftlas A. F., Hoover R. N., Brinton L. A., Szklo M., Olson D. R., Salane M., Wolfe J. N. (1991). Mammographic densities and risk of breast cancer.. Cancer.

[OCR_00623] Saftlas A. F., Szklo M. (1987). Mammographic parenchymal patterns and breast cancer risk.. Epidemiol Rev.

[OCR_00627] Saftlas A. F., Wolfe J. N., Hoover R. N., Brinton L. A., Schairer C., Salane M., Szklo M. (1989). Mammographic parenchymal patterns as indicators of breast cancer risk.. Am J Epidemiol.

[OCR_00636] Self S., Prentice R., Iverson D., Henderson M., Thompson D., Byar D., Insull W., Gorbach S. L., Clifford C., Goldman S. (1988). Statistical design of the Women's Health Trial.. Control Clin Trials.

[OCR_00643] Warner E., Lockwood G., Tritchler D., Boyd N. F. (1992). The risk of breast cancer associated with mammographic parenchymal patterns: a meta-analysis of the published literature to examine the effect of method of classification.. Cancer Detect Prev.

[OCR_00653] Whitehead J., Carlile T., Kopecky K. J., Thompson D. J., Gilbert F. I., Present A. J., Threatt B. A., Krook P., Hadaway E. (1985). Wolfe mammographic parenchymal patterns. A study of the masking hypothesis of Egan and Mosteller.. Cancer.

[OCR_00665] Wolfe J. N. (1976). Breast patterns as an index of risk for developing breast cancer.. AJR Am J Roentgenol.

[OCR_00661] Wolfe J. N. (1976). Risk for breast cancer development determined by mammographic parenchymal pattern.. Cancer.

[OCR_00669] Wolfe J. N., Saftlas A. F., Salane M. (1987). Mammographic parenchymal patterns and quantitative evaluation of mammographic densities: a case-control study.. AJR Am J Roentgenol.

